# Epidemiology of cancer in Colombia

**DOI:** 10.25100/cm.v49i1.3877

**Published:** 2018-03-30

**Authors:** Luis Eduardo Bravo, Nubia Muñoz

**Affiliations:** 1 Editor asociado, Revista Colombia Médica, Universidad del Valle, Cali, Colombia; 2 Professor Emeritus del Instituto de Cancerología de Colombia, Bogota, Colombia

In this special issue dedicated to cancer, Colombia Medica discloses an analysis of the cancer situation in Colombia and Ecuador. The analysis is based on the data collected and analyzed by several Population-based Cancer Registries (PBCR), carried out with an inter-institutional collaborative effort of public and private Colombian universities, Municipal and Provincial Secretaries of Health, and the Ministry of Health and Social Protection through the National Institute of Cancer of Colombia; and of the Fight Cancer Foundation of Ecuador, SOLCA-Core of Quito. This valuable contribution of the academic sector to the control and surveillance of cancer in Colombia needs reciprocity from the Ministry of Health. It is necessary to regulate the participation of RPC in the cancer information system; and to assign permanent resources to guarantee their sustainability.

For forty years the Population-based Cancer Registry of Cali (RPC-Cali) was the only source of valid information on the incidence of cancer in Colombia ^1^. To increase coverage, the National Cancer Institute of Colombia (NCI-Col), with the advisory of the Universidad del Valle, promoted during the first decade of the XXI century the creation of PBCR in strategic regions of the country. Thanks to this effort, the incidence information of the Colombian cities of Pasto, Manizales and Bucaramanga was added to that of Cali and published since 2012 in Cancer Incidence in Five Continents ^2^, and the four Colombian RPC participated in the CONCORD ^3^ study, the global monitoring program for cancer survival. Due to advances in cancer control and the great strength of its RPC, Cali is the first city in the world to implement the initiative "C/Can 2025: Challenge of Cities Against Cancer;" a project of the Union for International Cancer Control (UICC) that seeks to increase the coverage and quality of oncological care in cities with more than one million inhabitants in low and middle income countries ^4^.

In this issue of Colombia Medica, the PBCR-Cali describes the principles and methods used to analyze 50 years of incidence (1962-2012), 30 years of mortality (1984-2014) and 15 years of survival data (1995-2009) ^5^. Six Colombian PBC-Registries and one Ecuadorian PBC-Registry show the collection, classification and analysis of all new cancer cases and cancer deaths that occurred in Quito, Cali, Pasto, Bucaramanga, Manizales, Barranquilla and Medellín during the period 2008-2012 ^6^ ^-^ ^12^.

The Population-based Cancer Registries of Cali, Quito and Pasto have at least 15 years of good quality information and present valid results of cancer incidence and mortality trends in their respective populations ^6^ ^-^ ^8^. 

The PBCR-Cali ^7^, PBC-Manizales ^10^ and the hospital-based cancer registry (HBC-Registry) of the NCI-Colombia ^13^, the only HBC-Registry in the country, present survival data for the types of cancer with the highest morbidity in Colombia: prostate, breast, cervix, colon and stomach.

Data analysis shows that there is a significant decrease in the incidence and mortality rates of the infectious-related cancers and tobacco-related cancers; and an increase in the incidence rates of cancers related to early detection activities (breast, prostate, colon) and new diagnostic techniques (thyroid cancer) ^6^ ^-^ ^8^.

Barranquilla and Medellín report for the first-time data on the incidence of cancer ^11^ ^,^ ^12^. Barranquilla, a coastal city and the main economic center of the Colombian Caribbean region, shows the highest incidence rates of breast and cervix uteri cancer, while its rates for gastric cancer and all cancer sites are the lowest in Colombia. It is important to conduct specific investigations to determine if these differences are the result of including non-resident cases, duplication and/or information underreporting. 

Medellín, located in the Andean mountains of northwestern Colombia, is the second most populated city in the country, with particular demographic characteristics; it presents the incidence for cancers prioritized by the Ten-Year Plan for Cancer Control in Colombia. It should be noted that nearly 100% of new cases of cancer registered have morphological verification ^12^, so that the results correspond more to a Population Registry of Pathology. In this sense, the information on the risk of cancer in Medellín is most probably underestimated since it does not include cases with a clinical or imaging diagnosis and those whose only evidence of cancer is the death certificate. Therefore, the rates for Medellín are not comparable with those of other Colombian PBC-Registries.

The NCI-Colombia characterizes the current situation of the supply of oncological services in Colombia ^14^, and it demonstrates that the certification of cancer deaths in Colombia is of good quality ^15^. The NCI-Colombia uses the information from four cancer registers ^7^ ^-^ ^10^and the official mortality figures, to make valid estimates of cancer incidence for the entire country and for each one of the provinces in Colombia ^15^. The cancer risk estimates for Colombia will be more precise when the information from Barranquilla ^11^and Medellín ^12^can be included in the future.

**Figure 1:**
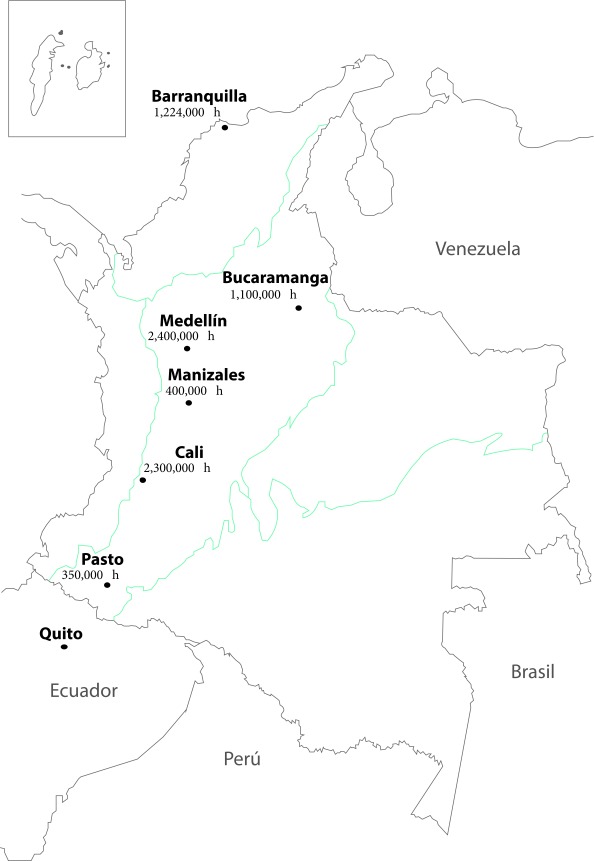
Location of the Population-based Cancer Registries in Colombia. The physical location of the RPC-Colombians is linked to their administrative dependency; all are in universities, except for the PBC-Antioquia, which is located in the Provincial Health Secretariat. The universities have been the main source of financial resources and of scientific and technical personnel for the PBC-Colombians; its directors have academic and research experience. Data are inhabitants

Figure 1 shows the location of the RPC-Colombians, and Table 1 the incidence rates for the five leading causes of cancer morbidity in Colombia, prioritized by the Ten-Year Plan for Cancer Control, 2012-2025.

**Table 1:** Rates of cancer incidence (100,000 persons-year) for the most frequent cancers, prioritized in the Ten-Year Plan for Cancer Control in Colombia.

Region	Breast C50	Prostate C61	Cervix C53	Colon C18-C21		Stomach C16	
				♂	♀	♂	♀
1. Cali	44.3	59.7	15.3	16.2	14.0	20.2	10.7
2. Pasto	27.7	27.3	18.0	8.4	9.0	26.7	11.8
3. Bucaramanga	41.2	40.9	13.0	14.3	13.7	17.1	10.2
4. Manizales	37.2	44.1	17.5	14.7	14.7	20.3	9.7
5. Barranquilla	65.7	43.0	26.6	9.6	9.8	4.4	2.2
6. Medellín	36.5	38.6	8.5	7.5	6.9	12.4	8.1
7. Colombia-INC	33.8	46.5	19.3	12.2	12.3	18.5	10.3
8. Quito-Ecuador	38.8	62.9	18.6	13.2	11.9	20.3	14.5

To estimate risk measures, PBC-Registries must have a delimited registry area and a clear definition of "case". In this definition, it is critical to include only the new cancer cases diagnosed in the permanent residents of the city and to exclude the cancer cases of patients referred to the city for diagnostic and/or treatment procedures. The data collection must be both passive and active in the different sources of information.

PBC-Registries require adequate and sustained resources to be successful. In Colombia, the per capita registration cost varies between US $0.05 to US $0.22. Between 20% and 45% of the total cost is due to activities with a fixed cost. Universities have been the main source of financial resources and of both scientific and technical personnel, which has allowed them to be successful. Another success factor of the PBC-Registries is the social recognition in the city, facilitating the process of data collection.

The report of NCI-Colombia ^14^shows this reality concerning oncological services in the country, the system serves 63,000 new cases of cancer annually ^16^. Colombia has 1,780 habilitated services, but only 25 providers offer joint chemotherapy, radiotherapy and surgery services. Nearly 50% of the offer is concentrated in Bogotá, and the provinces of Antioquia and Valle del Cauca; 87.8% is offered by Institutions, and 12.2% by independent (health) professionals. 66.7% of the oncology services are outpatient, 17.4% of diagnostic support and therapeutic complementation services, and 15.9% of surgical services; 87.9% of the offer of oncological services in Colombia is in the private sector. There is clear evidence of fragmentation in the provision, so it is necessary to redefine the services and to make a comprehensive oncological care approach for the diagnosis and treatment of patients with cancer, in order to improve clinical outcomes.

Despite this wide range of services, the 5-year global survival of childhood cancer in Cali (51%) is between 26% and 32% below the reported results for affluent countries (77% to 83%). This means that, if around 1,500 to 1,600 children with cancer are treated in Colombia each year, 765 to 816 die within 5 years after diagnosis. Of these, 390 to 512 deaths per year would be avoidable. This survival gap is maintained in all groups of neoplasms, except for Hodgkin's lymphoma (88% vs 95%). In adults, the situation is similar; the 5-year net survival to prostate, cervix and breast cancer is between 20 and 30 points below that observed in North America and Europe. During the period 2000-2004, the 5-year net survival improved for cancers of the breast, cervix, prostate, melanoma and thyroid, although in the period 2005-2009, it was observed a stagnation. In stomach, liver and lung cancer, the 5-year net survival was less than 15% ^7^.

The PBC-Manizales ^10^ analyzed the differences in the survival for breast, cervix, lung, prostate and stomach cancers; it highlights the existence of important inequities in cancer survival related to health insurance and socioeconomic status, attributable to the barriers and delays in obtaining diagnostic care that are associated with more advanced stages at the time of diagnosis. 

The HBC-Registry of NCI-Colombia ^13^, Colombia's only hospital-based cancer registry, analyzed survival in 1,928 cases of breast cancer and 1,189 cases of cervix uteri cancer. The estimated overall survival probability was 79.6% for breast cancer and 63.3% for cervix uteri cancer. Overall survival was 32.2% for stage IV breast cancer and 22.6% for stage IV cervical cancer. These survival estimates are like those reported by the cancer-registries ^4^ ^,^ ^7^ ^,^ ^10^; It would be expected that the survival estimates in a cancer center would be higher than the estimates observed by the cancer registries. It is necessary to review the guidelines for clinical management in cancer patients treated at the NCI-Colombia.

In Conclusion, the pioneer effort of the population-based Cancer registry of Cali and of the Universidad del Valle and their collaboration with other academic and public institutions has made possible a precise estimation of the cancer burden in various regions of Colombia and in Quito, Ecuador; this information is basic and essential in the planning of strategies for cancer control.
