# Supplementary material for: Epidemiology of cancer in Colombia
Source: Colomb Med (Cali). 2018 Mar 30;49(1):9–12. doi: 10.25100/cm.v49i1.3877 (PMC6018829; doi:10.25100/cm.v49i1.3877)
Supplement: Related texts

# Editorial: Epidemiologia del cáncer en Colombia

En este número especial dedicado al cáncer, Colombia Médica divulga un análisis de la situación de cáncer en Colombia y Ecuador. El análisis se basa en los datos recolectados y analizados por varios registros de cáncer, realizado con un esfuerzo colaborativo interinstitucional de universidades públicas y privadas colombianas, Secretarias Municipales y Departamentales de Salud y del Ministerio de Salud y la Protección Social a través del Instituto Nacional de Cancerología de Colombia; y de la Sociedad de Lucha contra el Cáncer del Ecuador, SOLCA-Núcleo de Quito. Este valioso aporte del sector académico al control y vigilancia del cáncer en Colombia necesita reciprocidad del Ministerio de Salud. Es necesario reglamentar la participación de los RPCs en el sistema de información de cáncer y asignar recursos permanentes para garantizar su sostenibilidad. 

Durante cuarenta años el Registro Poblacional de Cáncer de Cali (RPC-Cali) fue la única fuente de información válida de la incidencia de cáncer en Colombia ^1^. Para aumentar la cobertura, el Instituto Nacional de Cáncer de Colombia (INC-Col) con la asesoría de la Universidad del Valle, impulsó durante la primera década del siglo XXI la creación de RPCs en regiones estratégicas del país. Gracias a este esfuerzo la información de incidencia de las ciudades colombianas de Pasto, Manizales y Bucaramanga fue sumada a la de Cali y publicada en el 2012 en*Cancer Incidence in Five Continents* ^2^,* *y los cuatro RPCs colombianos participaron en * * el estudio CONCORD ^3^, el programa global para la vigilancia mundial de la supervivencia al cáncer. Por los avances en el control del cáncer y por la gran fortaleza del Registro de Cáncer, Cali es la primera ciudad del mundo en implementar la iniciativa “C/Can 2025: Desafío de Ciudades Contra el Cáncer”; un proyecto de la Unión Internacional Control del Cáncer (UICC) que busca aumentar la cobertura y la calidad de la atención oncológica en las ciudades seleccionadas de más de un millón de habitantes de los países de bajos y medianos ingresos ^4^. 

En este número de Colombia Medica, el RPC-Cali describe los principios y métodos utilizados para analizar 50 años de incidencia (1962-2012), 30 años de mortalidad (1984-2014) y 15 años de supervivencia (1995-2009) ^5^. Seis registros RPC colombianos y uno ecuatoriano, muestran la recopilación, clasificación y análisis de todos los casos nuevos y las defunciones por cáncer ocurridas en Quito, Cali, Pasto, Bucaramanga, Manizales, Barranquilla^ ^y Medellín durante el periodo 2008-2012 ^6^^-^^12^. 

Los registros de cáncer de Cali, Quito y Pasto disponen de al menos 15 años de información de buena calidad y presentan resultados válidos de la tendencia de la incidencia y mortalidad por cáncer en sus respectivas poblaciones ^6^^-^^8^. 

Los RPC-Cali ^(^^7^, RPC-Manizales ^10^y el Registro Hospitalario de Cáncer (RHC) del Instituto Nacional de Cancerología de Colombia ^13^, único RHC del país, presentan datos de supervivencia para los tipos de cáncer de mayor morbilidad en Colombia: próstata, mama, cuello uterino, colon y estómago. 

La evidencia muestra que hay descenso significativo de las tasas de incidencia y mortalidad de los tipos de cáncer relacionados con agentes infecciosos y el consumo del tabaco; y aumento en las tasas de incidencia de los canceres relacionados con actividades de detección temprana (mama, próstata, colon) y nuevas técnicas diagnósticas (cáncer de tiroides) ^6^^-^^8^.

Barranquilla y Medellín divulgan por primera vez datos sobre incidencia de cáncer ^11^^,^^12^. Barranquilla ciudad costera y el principal centro económico de la región caribe colombiana, muestra las tasas más altas de incidencia de cáncer de mama y cuello uterino al tiempo que las tasas para cáncer gástrico y todas las localizaciones de cáncer son las más bajas de Colombia^ ^. Es importante realizar investigaciones específicas para determinar si estas diferencias son resultado de incluir casos de no-residentes, de duplicidad y/o subregistro de información.

Medellín, segunda ciudad más poblada del país ubicada en las montañas andinas del noroeste colombiano, con características demográficas particulares, presenta la incidencia para los cánceres priorizados por el Plan decenal para el Control del Cáncer en Colombia. Debe destacarse que cerca del 100% de los casos nuevos de cáncer registrados tienen verificación morfológica ^12^, por lo que los resultados corresponden más a un Registro Poblacional de Patología. En este sentido, la información del riesgo de cáncer en Medellín debe estar subestimada al no incluir los casos con diagnóstico clínico o por imágenes y aquellos que tienen como única evidencia de cáncer el certificado de defunción. Por lo tanto, las tasas de Medellín no son comparables con las de los otros RPCs colombianos.

El INC-Col caracteriza la situación actual de la oferta de servicios oncológicos en Colombia ^14^y demuestra que la certificación de las defunciones por cáncer en Colombia es de buena calidad ^15^. El INC-Col utiliza la información de cuatro registros de cáncer ^7^^-^^10^^)^ y las cifras oficiales de mortalidad, para hacer estimaciones válidas de la incidencia de cáncer para todo el país y para cada uno de los departamentos de Colombia ^15^. Las estimaciones del riesgo de cáncer para Colombia serán más precisas cuando en el futuro se pueda incluir la información de Barranquilla ^11^y Medellín ^12^.

La Figura 1 muestra la localización de los RPC-colombianos y la Tabla 1 las tasas de incidencia para las cinco primeras causas de morbilidad por cáncer en Colombia priorizadas por el Plan Decenal para el Control del Cáncer, 2012-2025.

**Figura 1:** Localización de los Registros Poblacionales de Cancer en Colombia. La ubicación física de los RPC-colombianos está ligada a su dependencia administrativa, todos están situados en universidades, con excepción del RPC-Antioquia ubicado en la Secretaria de Salud Departamental. Las universidades han sido la principal fuente de recursos financieros y de personal científico y técnico de los RPC-colombianos; sus directores tienen experiencia académica y en investigación. Datos son el número de habitantes

**Tabla 1:** Tasas de incidencia de cáncer (100,000 personas-año) para los canceres más frecuentes, priorizados en el Plan Decenal Para el control del Cáncer en Colombia.

Región	Mama C50	Próstata C61	Cérvix C53	Colon C18-C21		Estómago C16	
				♂	♀	♂	♀
1. Cali	44.3	59.7	15.3	16.2	14.0	20.2	10.7
2. Pasto	27.7	27.3	18.0	8.4	9.0	26.7	11.8
3. Bucaramanga	41.2	40.9	13.0	14.3	13.7	17.1	10.2
4. Manizales	37.2	44.1	17.5	14.7	14.7	20.3	9.7
5. Barranquilla	65.7	43.0	26.6	9.6	9.8	4.4	2.2
6. Medellín	36.5	38.6	8.5	7.5	6.9	12.4	8.1
7. Colombia-INC	33.8	46.5	19.3	12.2	12.3	18.5	10.3
8. Quito-Ecuador	38.8	62.9	18.6	13.2	11.9	20.3	14.5

Para estimar la incidencia de cáncer, los RPC deben tener un área de registro delimitada y una definición clara de “caso”. En esta definición es crítico incluir solo los casos nuevos diagnosticados en los residentes permanentes de la ciudad; y excluir los casos de pacientes remitidos a la ciudad para procedimientos de diagnóstico y/o tratamiento. La recopilación de datos debe ser tanto pasiva como activa en las diferentes fuentes de información.

Los RPC requieren recursos adecuados y sostenidos para lograr ser exitosos. En Colombia, el costo del registro por habitante varía entre US $ 0.05 a US $ 0.22. Entre el 20% y el 45% del costo total se debe a actividades con costo fijo. Las universidades han sido la principal fuente de recursos financieros y de personal tanto científico como técnico que les ha permitido ser exitosos. Otro factor de éxito de los RPC es el reconocimiento social en la ciudad facilitando así el proceso de recopilación de datos.

En Colombia se ha consolidado una gran oferta de servicios oncológicos. El reporte del INC-Col^14^muestra esta realidad en el país, el sistema atiende los 63,000 casos nuevos anuales de cáncer ^16^. Tiene 1,780 servicios habilitados, pero sólo 25 prestadores ofertan de forma conjunta los servicios de quimioterapia, radioterapia y cirugía. Cerca del 50% de la oferta se concentra en Bogotá, Antioquia y Valle del Cauca. El 87.8% es ofrecido por Instituciones y el 12.2% por profesionales independientes. El 66.7% de los servicios oncológicos son de consulta externa, el 17.4% de servicios de apoyo diagnóstico y complementación terapéutica y el 15.9% de servicios quirúrgicos. El 87.9% de la oferta de servicios oncológicos en Colombia está en el sector privado. Hay claras evidencias de fragmentación en la prestación, por lo que es necesario redefinir los servicios y hacer un enfoque de atención oncológica integral para el diagnóstico y tratamiento de los pacientes con cáncer; con el fin de mejorar los resultados clínicos.

A pesar de esta amplia oferta de servicios, la supervivencia global a 5 años del cáncer infantil en Cali (51%) está entre 26% y 32% por debajo de los resultados reportados en países afluentes (77% a 83%). Esto significa, que, si en Colombia se tratan alrededor de 1,500 a 1,600 niños con cáncer al año, 765 a 816 mueren en los 5 años posteriores al diagnóstico. De estos, 390 a 512 muertes anuales serían evitables. Esta brecha de supervivencia se mantiene en todos los grupos de neoplasias, excepto en el linfoma de Hodgkin (88% vs 95%). En adultos la situación es similar y la supervivencia neta a 5 años al cáncer de próstata, cuello uterino y mama esta entre 20 y 30 puntos por debajo a la observada en Norteamérica y Europa. Durante el periodo 2000-2004, la supervivencia neta a 5 años mejoró para los cánceres de mama, cuello uterino, próstata, melanoma y tiroides, aunque en el periodo 2005-2009 se observó un estancamiento. En cáncer de estómago, hígado y pulmón, la supervivencia neta a 5 años fue inferior al 15% ^7^.

El RPC-Manizales ^10^analizó las diferencias en la supervivencia del cáncer de mama, cuello uterino, pulmón, próstata y estómago y pone en evidencia la existencia de inequidades importantes en la supervivencia del cáncer relacionadas con el seguro de salud y la posición socioeconómica, atribuibles a las barreras y retrasos en la obtención de atención de diagnóstico que se asocian con etapas más avanzadas en el momento del diagnóstico.

El registro hospitalario del INC-Colombia ^13^, único registro hospitalario de cáncer de Colombia, analizó la supervivencia en 1,928 casos de cáncer de mama y 1,189 casos de cáncer de cuello uterino. La probabilidad de supervivencia global estimada fue del 79.6% para cáncer de mama y del 63.3% para cáncer de cuello uterino. La supervivencia fue del 32.2% para el cáncer de mama en estadio IV y del 22.6% para el cáncer de cuello uterino en estadio IV. Estas estimaciones de supervivencia son similares a las informadas por los registros de cáncer ^4^^,^^7^^,^^10^; Se esperaría que las estimaciones de supervivencia en un centro de cáncer serían más altas que las estimaciones observadas por los registros de cáncer. Es necesario revisar las pautas para el manejo clínico en pacientes con cáncer tratados en el INC-Colombia 

En Conclusión, el esfuerzo pionero del Registro Poblacional de Cáncer de Cali y de la Universidad del Valle y su colaboración con otras entidades académicas y públicas han hecho posible una estimación precisa de la carga de cáncer en varias regiones de Colombia y en Quito, Ecuador; esta información es básica y esencial en el planteamiento de estrategias para el control del cáncer.
